# Climate change opportunities reduce farmers' risk perception: Extension of the value-belief-norm theory in the context of Finnish agriculture

**DOI:** 10.3389/fpsyg.2022.939201

**Published:** 2022-08-24

**Authors:** Jaana Sorvali, Xing Liu, Janne Kaseva

**Affiliations:** Natural Resources Institute Finland (Luke), Helsinki, Finland

**Keywords:** climate change, opportunity, farmer, agriculture, value-belief-norm theory, survey, Finland

## Abstract

Global agriculture faces severe challenges due to climate change. For boreal agriculture, climate change might also bring opportunities as the growing season lengthens, if the risks of climate change are managed properly. Agricultural production is a source of greenhouse gases, while agricultural land has also a great possibility to mitigate climate change as a carbon sink. Farmers are the central group for implementing these actions. Their views and beliefs contribute to their corresponding pro-environmental agricultural behavior. This research is based on the theory of value-belief-norm (VBN) as a predictive model of pro-environmental agricultural behavior. We extend the theory by studying how opportunities caused by climate change affect pro-environmental behavior in agriculture and present differences between farmer groups and experiment with the longitudinal possibilities of the theoretical model. Based on the structured survey responses from 4,401 farmers in Finland in 2018 and 2000 responses in 2020, we found that all the elements of VBN theory did help to predict intention for climate change mitigation, among which felt possibility to perform mitigation practices was the strongest predictor while risk perception was rather an unimportant one. Furthermore, opportunities caused directly or indirectly by climate change have an effect on Finnish farmer's implementation of mitigation practices. Therefore, future efforts in agricultural research and policy in Finland should concentrate to bring forth concrete farm-level mitigation practices with proven environmental benefits and the direct and indirect opportunities should be given more attention.

## Introduction

Strong scientific proof supports anthropogenic climate change and shows that the pace of change has accelerated. Mitigation actions have not yet been able to curb emission trends globally (IPCC, 2021). Agriculture constitutes around 10% of the greenhouse gas (GHG) emissions in Europe without land use, land use change, and forestry (LULUCF) sector emissions; when these are included, agricultural emissions are around 20% of Europe's total emissions (EEA, 2019; YM, 2021). In Finland, the agricultural GHG emissions follow the Europe's average. The Finnish government has committed to reduce agricultural-related emissions by 29% by 2035 (MMM, 2022).

Climate change brings challenges to Finnish agriculture (Hakala et al., 2011; Tao et al., 2020). New crop diseases and pests and their earlier occurrence can be expected (Peltonen-Sainio and Jauhiainen, 2020). The increased frequency of extreme weather conditions, such as increased precipitation, heavy rainfall (Peltonen-Sainio et al., 2021), and drought, further challenges field drainage and water retention (Peltonen-Sainio et al., 2016). More frequent drought and heat stress with other environment-related constraints may lead to higher yield variability and yield loss (Purola et al., 2017). Climate change may also challenge both nutrient and carbon cycles because warming may speed up both the production and decomposing of organic matter. In addition, changes in the water cycle impact on the leaching of organic matter and nutrients from soil to water (MMM, 2014).

While the negative impacts of climate change are already felt globally (Gammans et al., 2017; Cianconi et al., 2020; IPCC, 2022), the northward shift of warmer climate might create opportunities for the boreal region as to their suitability for agriculture in terms of new species and potential yield increase (King et al., 2018; Unc et al., 2021). These opportunities are also applicable to Finland (Peltonen-Sainio et al., 2018, 2020) and are recognized by the farmers (Sorvali et al., 2021). Even if high-latitude agriculture may benefit from climate change, it still needs to contribute to reduction of GHG emissions as defined in national and international climate policies and agreements. Naturally, farmers are the central group for implementing these climate actions at the farm level (Gomes and Reidsma, 2021).

Farmers' engagement in climate change mitigation requires awareness of climate change (Arbuckle et al., 2013a), and personal values have also been found to play a role (Sorvali et al., 2022). Finnish farmers have a very high awareness of climate change, but they have differing views on the balance of risks and opportunities, although especially opportunities are highly recognized. Similarly, farmers' views on their responsibilities to act as do their perception of own possibilities to adapt and mitigate differ in different farmer groups (Sorvali et al., 2021). Finnish farmers are, in general terms, strongly inclined toward self-transcendence values and universalism values (Sorvali et al., 2022), which are especially connected to pro-environmental concerns (Hansla et al., 2008).

Without understanding the farmers' values, beliefs, and normative views, it is impossible to draft policy measures that will be accepted and thoroughly implemented by farmers. In this article, we extend the study of Sorvali et al. (2021, 2022) and apply the theory of value-belief-norm (VBN) (Stern, 2000) to deepen the knowledge of Finnish farmers pro-environmental behavior. We contribute to previous psychological literature by bringing in perceived opportunities caused by climate change as a new predictive element. Furthermore, we aim to compare the results between different farmer groups and between two spaces in time (2018 and 2020). Thus, we directly contribute to the VBN theory with the new element of opportunity and longitudinal data in which the participants were identical.

The rest of the chapters are organized as follows: Chapter two gives a short overview of the VBN theory, the terminology, and an overview of previous research with specific emphasis on agriculture. In chapter three, materials and methods are described, and chapter four presents the results. The findings are being discussed in chapter five together with limitations of the research and suggestions for future research and concluding remarks are presented in chapter six.

## VBN and its applications in pro-environmental behavior

**Figure 1:**
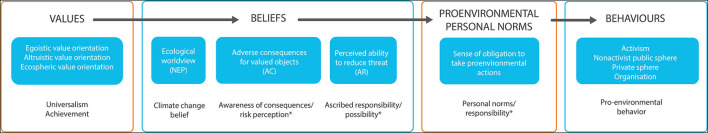
Original VBN theory (Stern, 2000) is shown in blue boxes. Under the boxes, corresponding terms are used in literature. Terms marked in asterisk are used in this research.

Theory of value-belief-norm (VBN) (Stern, 2000) conceptualizes how certain values, pro-environmental worldview (NEP), understanding of the consequences of environmental problems (AC) together with perceived ability to reduce threat (AR), and personal norms (PN) explain pro-environmental behavior. The VBN theory is based on the basic human values (Schwartz, 1992), the Norm-Activation Model (NAM) (Schwartz, 1977; Schwartz and Howard, 1981), and the New Environmental Paradigm (NEP) (Dunlap and Van Liere, 1978) theories. As VBN theory or parts of it has widely been used in studying environmental behaviors in different contexts by scholars from differing backgrounds, the terminology has become incoherent and sometimes difficult to follow. Thus, an effort is made here to connect the original VBN terminology to the terminology used in similar fashion in different contexts and simplify the terminology (Figure 1).

*Values* are “desirable trans-situational goals, varying in importance, that serve as guiding principles in the life of a person or other social entity” (Rokeach, 1973; Schwartz, 1994). Values have been thoroughly studied in the literature concerning environmental behavior, and especially self-transcendence values (universalism and benevolence) have been related to high environmental concern (Stern, 2000; Schultz et al., 2005; Hansla et al., 2008). The connection between values and behavior has been widely debated, but it is commonly accepted that values predict different attitudes and behaviors (Sagiv et al., 2017). Research on farmers' values has also gained attention in recent years, and self-transcendence values have been found to score high among farmers in different countries (Dobricki, 2011; Baur et al., 2016; Sorvali et al., 2022).

A *belief* is one's personal knowledge on how things are. A belief can be scientifically correct or incorrect. What is important is that person believes it to be true (Heberlein, 2012). Farmers' beliefs connected with the human–environment interaction in the climate change context have been the growing focus of research in the past decade (Karki et al., 2020) with many examples around the world (USA: Arbuckle et al., 2013a,b; Australia: Buys et al., 2012; Chile: Roco et al., 2015; New Zealand: Niles et al., 2016; Italy: Nguyen et al., 2016; Sweden: Asplund, 2016; South Africa: Hitayezu et al., 2017; Bangladesh: Kabir et al., 2017; Nepal: Khanal et al., 2018; Norway: Brobakk, 2018; Peru: Altea, 2020 and Finland: Peltonen-Sainio et al., 2020; Sorvali et al., 2021). Perception is sometimes used interchangeably with attitude or belief, but it is a broader term incorporating knowledge, beliefs, attitude, concern, affect, and perceived risk (Whitmarsh and Capstick, 2018).

Awareness of adverse consequences for valued objects (awareness of consequences) essentially means belief that environmental conditions, such as climate change, threaten something that the individual values (Steg et al., 2005). In literature, *risk perception* has been used interchangeably with awareness of consequences (e.g., Arbuckle et al., 2015), and it has been identified in many studies as important predictor of pro-environmental action (Zheng et al., 2020; Maartensson and Loi, 2022). Risk is a situation, event, or activity which might lead to uncertain and adverse outcomes toward something of value (Böhm and Tanner, 2019). According to IPCC (2018), climate change risk “refers to the potential for adverse consequences of a climate-related hazard, or of adaptation or mitigation responses to such a hazard, on lives, livelihoods, health and wellbeing, ecosystems and species, economic, social and cultural assets, services (including ecosystem services), and infrastructure”. Thus, risk perception refers to the subjective judgment of people about those climate change risks (IPCC, 2018). Risk perceptions of climate change vary internationally (Lee et al., 2015) and change over time (Milfont et al., 2017). Farmers' climate change-related risk perceptions in differing geo-political and climate conditions have been a rising area of interest for research during recent years (Niles, Lubell and Haden, 2013; Eitzinger et al., 2018).

Perceived ability to reduce threat, or more simply, felt *possibility* to perform pro-environmental behavior that will, in our case, reduce the effects of climate change, is related to self-efficacy that means “a judgment of one's capability to accomplish a certain level of performance” (Bandura, 1986). Social norms are behavioral regularities, or rules and standards, that come with sanctions if they are not followed (Heberlein, 2012; Keizer and Schultz, 2019). *Personal norms* are rules or standards for one's own behavior (Kallgren et al., 2000). Any behavior that has an impact on the environment is considered environmental behavior (Gatersleben, 2019). *Pro-environmental behavior* (or environmentally friendly; ecological; conservation behavior) can be defined as either goal-directed: “behavior that consciously seeks to minimize the negative impact of one's actions on the natural and built world”. (Kollmuss and Agyeman, 2002), or non-goal-directed: “behavior that harms the environment as little as possible or even benefits the environment” (Steg and Vlek, 2009).

The VBN theory has been widely used to explain pro-environmental behavior in different contexts, such as in sustainable consumption (Han et al., 2020; Yakut, 2021; Ahn and Kwon, 2022), sustainable tourism (Han, 2015, 2021; Manosuthi et al., 2020; Sharma and Gupta, 2020), sustainable management of protected marine areas (Wynveen et al., 2015), and different geographical and ethnic contexts (Chen, 2015; Ghazali et al., 2019; Medina et al., 2019). Although thorough VBN applications for agriculture are still few in numbers, the separate VBN elements have been included in many studies in the agricultural sector, for example, connections between climate change beliefs to adaptation and mitigation action in Denmark (Jørgensen and Termansen, 2016), and climate change beliefs and norms in Scotland (Barnes and Toma, 2012) and Germany (Eggers et al., 2015; Jantke et al., 2020). From the more thorough applications of the VBN theory in agriculture, Price and Leviston (2014) found that Australian farmers' context and social-psychological characteristics predict their pro-environmental land management practice, further validating arguments that agricultural change is driven by individual motivations. Sanderson et al. (2018) draw upon the VBN theory and the result indicated that value-based engagement strategies could provide significant leverage to increase public participation regardless of farming or non-farming participating groups. With the VBN framework, Rezaei-Moghaddam et al. (2020) analyzed Iranian farmers' pro-environmental behaviors concerning the adoption of clean technology of local compost. The results found that strong responsibility for nature increases adoption of the pro-environmental behavior. Zhang et al. (2020) compare the predictive power of the VBN theory with the Theory of Planned Behavior (TPB) in the context of climate change mitigation and adaptation in China. The study provided evidence that the VBN theory performed better when explaining mitigation behaviors of Chinese farmers.

Climate change opportunities are defined here as direct opportunities caused by global warming (e.g., possibility to extend agricultural production to more northern areas because of the rise in average temperatures) and as indirect opportunities where an action becomes beneficial because of the need to adapt or to mitigate climate change (e.g., development of new technology). According to Finnish farmers, climate change will bring opportunities for agriculture through, for example, longer growing seasons and bigger yields. Moreover, the farmers' perception of opportunities has grown during the years (Sorvali et al., 2021). Sorvali et al. (2021) also suggest that farmers who believe strongly in climate change opportunities also believe in their possibilities to adapt to future conditions and do not feel responsible for mitigation. As Finnish farmers' value profiles (Sorvali et al., 2022) and climate perceptions vary significantly between different groups (most notable differences were found between genders, age groups, and farming systems) (Sorvali et al., 2021), the connections and their impacts to pro-environmental behavior, such as mitigation willingness, needed more thorough studying. Research on climate change opportunities is scarce, and the psychological effects of existing climate change opportunities to pro-environmental behavior have not been studied at all.

Therefore, our hypotheses are (1) the VBN theory elements form a causal path from pro-environmental value of universalism *via* climate change belief, risk perception, felt possibility to mitigate, and felt responsibility to mitigate to pro-environmental behavior as suggested by Stern (2000), (2) opportunities have negative direct effect to risk perception and felt responsibility to mitigate climate change and pro-environmental behavior based on previous research by Sorvali et al. (2021), and (3) different elements of the VBN theory are highlighted in different farmer groups: gender (men/women), age (under 40/40 years or older), and farming method (organic/conventional) and at different years (2018 and 2020) as suggested in Sorvali et al. (2021, 2022).

## Materials and methods

A standardized survey was sent *via* e-mail in 2018 to all Finnish farmers. The participants were asked to answer over 200 structured statements concerning their climate change beliefs, and their views on adaptation and mitigation practices in agriculture (described in detail in Peltonen-Sainio et al., 2020; Sorvali et al., 2021). Values and future views were also asked. A follow-up survey was sent in 2020 to those who answered the first survey. In 2018, 4,401 farmers answered the survey (12% of all the Finnish farmers with an available e-mail address and 9% of all the farmers in Finland), and in 2020, 2,000 farmers (45%) answered the follow-up survey. The respondents represented the Finnish farming community well (see detailed descriptions of the respondents in Appendix 1). The basic data used here are the 2020 survey data, but differences to the corresponding statements in 2018 are also studied.

Farmers' values were studied using the refined Theory of Basic Human Values by Schwartz et al. (2012). The original theory of Basic Human Values was based on the idea of 10 basic values that form a circular continuum of motivations (Schwartz, 1992). Later, the theory was refined to comprise 19 basic values (Schwartz et al., 2012). Finnish farmers' values and the methodology used are reported in Sorvali et al. (2022). In the original VBN theory, an earlier version of the Schwartz value scale is used, where biospheric values correspond to universalism values, altruistic values to benevolence values, and egoistic values to hedonism. Universalism has the strongest positive connection to the other VBN elements and achievement to the climate change opportunities (Sorvali et al., 2022) and was therefore included in this study. Values were measured only in the 2018 survey, but as values stay relatively stable over time (Sagiv et al., 2017) and the same respondents were studied in both 2018 and 2020 surveys, it was safe to use the 2018 measured values also in the 2020 data analysis.

Instead of the NEP scale that measures general environmental attitudes and is used in the original VBN theory, a more climate change-specific measure was needed. Thus, climate change belief was used and it was measured by a scale differentiating between anthropogenic climate change and climate change caused by nature's own processes (Arbuckle et al., 2015). Risk perception was measured with a single statement relating to the climate change risks to Finnish agriculture. Climate change opportunities were measured with six statements covering different levels of agricultural actors in Finland and possibility to influence mitigation with five statements. Felt responsibility for mitigation was measured with two statements and pro-environmental behavior with one statement about the farmer's intended mitigation action. Although criticism has been presented against studying intention as a prediction for actual behavior also in the agricultural setting (Niles et al., 2016), for this research, the measures for actual mitigation behavior were not possible to obtain as there are no sufficient documented data on mitigation action undertaken by farmers in Finland. Statements and response scales are presented in Appendix 2, and correlations and other statistical variables are presented in Table 1. Pro-environmental behavior was not asked similarly in the 2018 survey and therefore also not reported here.

**Table 1:** Spearman's correlations, means, standard deviations, skewness, and kurtosis of the variables for climate change belief, risk perception, opportunity, possibility, personal norms, pro-environmental behavior, achievement, and universalism values used in path models.

	**Achievement**	**Universalism**	**Climate belief**	**Opportunity**	**Risk perception**	**Possibility**	**Responsibility**	**Pro-environmental behavior**
Achievement	1	0.527	^***^	^***^	^**^	0.425	0.055	0.085
Universalism	−0.01	1	^***^	0.052	^***^	^***^	^***^	^***^
Climate belief	−0.10	0.28	1	^***^	^***^	^***^	^***^	^***^
Opportunity	0.16	−0.04	−0.11	1	^***^	^***^	0.871	0.376
Risk perception	−0.06	0.15	0.29	−0.37	1	^***^	^***^	^***^
Possibility	0.02	0.31	0.39	0.10	0.21	1	^***^	^***^
Responsibility	−0.04	0.29	0.38	0.00	0.24	0.47	1	^***^
Pro–environmental behavior	−0.04	0.27	0.37	−0.02	0.15	0.48	0.38	1
**Mean**	3.49	4.38	4.06	3.25	2.99	3.46	3.38	2.32
**SD**	0.92	0.81	0.78	0.7	1	0.85	0.58	0.63
**Skewness**	−0.07	−0.5	−0.68	−0.18	0.04	−0.54	−0.04	−0.36
**Kurtosis**	−0.14	0.6	0.78	0.64	−0.33	0.39	0.04	−0.68
**Cronbach's** **α**	0.64	0.87	-	0.8	-	0.75	0.76	-

*Statistical significance (H_0_:|r|=0):*

** *p <0.01, and*

*** *p <0.001*.

The path model was selected for analyzing the relations between the sum variables formed, allowing to test all the direct and indirect effects of the variables simultaneously. Spearman's rho was applied to test correlations between the variables because half of the variables were measured in ordinal scale. The internal consistency of the sum variables was tested using Cronbach's alpha and was found acceptable in all cases. The same theory-based path model was fitted also for different farmer groups according to gender (men/women), age (under 40/40 years or older), and farming method (organic/conventional). The models for 2 years (2018/2020) were also compared, although the answers for one statement concerning pro-environmental behavior were not gathered for both years.

Despite a few ordinal scale variables, large sample size and normally distributed variables enabled the use of maximum likelihood (ML) estimation. Modification indices, such as Lagrange's multiplier test, were used to evaluate the models, but only in one case, a new significant relation that improved the model was included. Goodness-of-fit models were evaluated by three different criteria and the chi-square test, which, however, are known to be problematic with large samples (Vandenberg, 2006). The indices used to assess model fit were the root mean square error of approximation (RMSEA) and the standardized root mean square residual (SRMR), in which values below 0.08 can be considered a reasonable fit and RMSEA ≤ 0.05 a good fit. In addition, we used the comparative fit index (CFI), in which values >0.90 can be considered as a reasonable fit and values over 0.95 a good fit (Hu and Bentler, 1999). SAS Enterprise Guide 7.15 (SAS Institute, Inc., Cary, NC, USA) was used in the statistical analyses.

## Results

### The base model for all farmers in 2020

**Figure 2:**
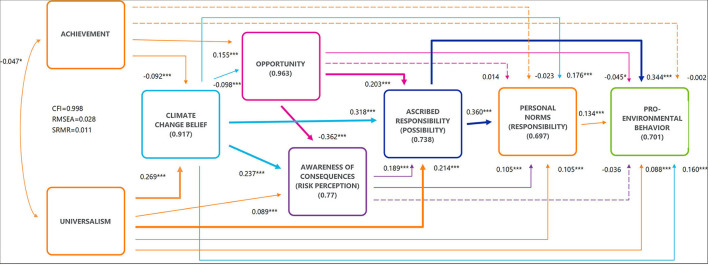
Path model for all farmers in 2020 (The base model, *N* = 1,965). The value on the double-headed arrow is the correlation coefficient, single-headed arrows are the standardized regression coefficients, and the values in parentheses are the standardized error variances. All the parameters of relations described by solid lines differed from zero, and the relations described by dashed lines were not statistically significant. Three goodness-of-fit indices are shown in the left: CFI, comparative fit index; CI, confidence interval; RMSEA, root mean square error of approximation; SRMR, standardized root mean square residual. Statistical significance (H0:|r|=0): * *p* < 0.05, ** *p* < 0.01 and *** *p* < 0.001.

The model fit for *year 2020* was good according to the indices (CFI = 0.998; RMSEA = 0.028; and SRMR = 0.011) (Figure 2). All the relations were statistically significant, except the paths from achievement to responsibility and to pro-environmental behavior, from opportunity to responsibility, and from risk perception to pro-environmental behavior (Figure 2; Table 2). These connections were, however, left into the model because of the aim to study how opportunity affects the models.

**Table 2:** Path model results for all farmers in 2020 (The base model, *N* = 1,965), women 2020 (*N* = 217), men 2020 (*N* = 1,748), farmers under 40 years old (*N* = 356), farmers 40 years old or older (*N* = 1,609), organic farmers (*N* = 239), conventional farmers (*N* = 1,694), and all farmers in 2018 (*N* = 1,965).

		**Estimates**							
**Variable**	**Predictor**	**All 2020**	**Women**	**Men**	**Under 40**	**40 or over**	**Organic**	**Conventional**	**All 2018**
Climate belief	Achievement	−0.092***	−0.087	−0.088**	−0.025	−0.108***	−0.051	−0.092***	−0.079***
Climate belief	Universalism	0.269***	0.179**	0.267***	0.336***	0.250***	0.457***	0.233***	0.270***
Opportunity	Achievement	0.155***	0.041	0.163***	0.198***	0.151***	0.187**	0.153***	0.160***
Opportunity	Climate belief	−0.098***	−0.212**	−0.074**	−0.135**	−0.091**	−0.118^o^	−0.089***	−0.024
Risk perception	Climate belief	0.237***	0.137*	0.246***	0.286***	0.224***	0.224***	0.236***	0.274***
Risk perception	Universalism	0.089***	0.040	0.085**	0.135**	0.078**	0.176**	0.073***	0.090***
Risk perception	Opportunity	−0.362***	−0.424***	−0.351***	−0.305***	−0.375***	−0.310***	−0.368***	−0.336***
Possibility	Opportunity	0.203***	0.161*	0.205***	0.123**	0.224***	0.154**	0.222***	0.239***
Possibility	Risk perception	0.189***	0.250**	0.182***	0.159**	0.197***	0.201***	0.189***	0.172***
Possibility	Universalism	0.214***	0.257***	0.210***	0.172**	0.223***	0.347***	0.196***	0.266***
Possibility	Climate belief	0.318***	0.313***	0.319***	0.414***	0.298***	0.223***	0.324***	0.260***
Responsibility	Opportunity	0.014	0.124	0.004	−0.006	0.021	−0.022	0.014	0.007
Responsibility	Risk perception	0.105***	0.146*	0.099***	0.063	0.117***	0.146*	0.096***	0.043*
Responsibility	Possibility	0.360***	0.234**	0.378***	0.433***	0.340***	0.282***	0.374***	0.534***
Responsibility	Climate belief	0.176***	0.214**	0.171***	0.196**	0.117***	0.279***	0.158***	0.206***
Responsibility	Universalism	0.105***	0.170**	0.089***	0.061	0.117***	0.033	0.120***	0.164***
Responsibility	Achievement	−0.023	0.008	−0.023	0.047	−0.043*	0.056	−0.034^o^	−0.037*
Pro-environmental behavior	Responsibility	0.134***	0.138*	0.132***	0.209**	0.118***	0.091	0.144***	**-**
Pro-environmental behavior	Opportunity	−0.045*	0.032	−0.053*	−0.019	−0.056*	−0.136*	−0.028	-
Pro-environmental behavior	Risk perception	−0.036	−0.099	−0.030	−0.052	−0.031	−0.112^o^	−0.024	-
Pro-environmental behavior	Possibility	0.344***	0.494***	0.330***	0.234***	0.366***	0.329***	0.332***	-
Pro-environmental behavior	Climate belief	0.160***	0.127*	0.163***	0.196**	0.153***	0.294***	0.148***	-
Pro-environmental behavior	Universalism	0.088***	0.059	0.085***	0.062	0.092***	0.058	0.088***	-
Pro-environmental behavior	Achievement	−0.002	−0.040	0.007	0.066	−0.017	−0.059	0.002	-
	**SRMR**	0.0108	0.0134	0.0096	0.0282	0.0082	0.012	0.013	0.032
	**RMSEA**	0.0279	0	0.0222	0.0828	0.0138	0	0.034	0
	**CFI**	0.9983	1	0.9989	0.9874	1	1	0.997	1
	$\chi_{df}^{2}$	2,774_28_	314_28_	2,406_28_	610_28_	2,212_28_	449_28_	2,275_28_	2,869_21_

Negative connections are marked in red, and connections >|±0.200| are bolded. Non-significant connections are highlighted with gray. Statistical significance (H_0_:|β|=0): ^o^0.05 < p <0.10, *p <0.05, **p <0.01, and ***p <0.001. The three goodness-of-fit indices and chi-square value are shown below.

The *values* of universalism and achievement correlated negatively (β = −0.047^*^) with each other in accordance with the basic human values theory. As hypothesized, *universalism* had a statistically significant direct effect to risk perception (β = 0.089^***^), but 45% of its effect was mediated *via* belief of climate change to risk perception (Appendix 3).

Universalism had also a direct effect to possibility to mitigate climate emissions (β = 0.214^***^), but again, 34% of its effect was mediated *via* climate change belief, opportunity, and risk perception. Universalism affected directly also responsibility for mitigation (β = 0.105^***^) and the pro-environmental behavior (β = 0.088^***^), with indirect effects at 63 and 68%, respectively. *Achievement* had a negative effect to climate change belief (β = −0.092^***^), indicating that higher achievement score led to a more questionable belief of the anthropogenic origin of climate change. A positive direct effect from achievement to opportunity was found (β = 0.155^***^), and only 6% of the total effect was mediated *via* climate change belief.

*Climate change belief* had a positive direct effect to felt responsibility of mitigation (β = 0.176^***^) and to pro-environmental behavior (β = 0.160^***^). *Opportunity* had a strong negative connection to risk perception (β = −0.362^***^), indicating that climate change opportunities reduce the notion of risk caused by climate change to agriculture. On the contrary, opportunity was positively connected to possibility to mitigate climate change (β = 0.203^***^). Opportunity had also a negative direct effect to pro-environmental behavior (β = −0.045^*^) that can be understood as unwillingness to mitigate something that will seemingly bring benefits. *Risk perception* had direct effects to mitigation possibility (β = 0.189^***^) and to responsibility of mitigation (β = 0.105^***^), but surprisingly not to intended action. Felt *possibility* to mitigate had strong direct effects to *responsibility* (β = 0.360^***^) and to *pro-environmental behavior* (β = 0.344^***^), indicating that felt responsibility mediated only 12% of the effect of felt possibility to pro-environmental behavior.

### Models for different farmer groups

The path models for different farmer groups showed also good or at least acceptable fit. The standardized regression coefficients for each farmer group are presented in Table 2, and the biggest differences are described below. The model for *men* showed no significant differences to the base model for 2020. The models for *farmers 40 years or older* and for *conventional farmers* were also similar as the base model (see Table 2). This was because male, 40 or older, and conventional farmer respondents formed such a large part of the whole sample (89, 82, and 84%, respectively) (Appendix 1). The model for *all farmers 2018* did not include the variable of pro-environmental behavior, as it was not included in the first survey. The relation from climate change belief to opportunity lost its statistical significance in the 2018 model compared to the 2020 one, but otherwise significant differences were not found.

In the model for *women*, two relations (from achievement to responsibility and from opportunity to pro-environmental behavior) turned from negative to positive compared to the base model, although these relations were no longer statistically significant (Table 2). For the model for women, many statistically significant relations from the values turned non-significant compared to the base model, from achievement to climate change belief (β = −0.087) and to opportunity (β = 0.041), and from universalism to risk perception (β = 0.040) and to pro-environmental behavior (β = 0.059). As a result, achievement became obsolete in the model altogether. The direct effects from universalism to climate change belief (β = 0.179^**^), to possibility (β = 0.257^***^), and to responsibility (β = 0.170^*^) were still in place. This would imply that achievement value has no effect to women's pro-environmental behavior and values alone do not predict pro-environmental behavior as much for women, as for the whole sample of farmers. The relation from climate change belief to opportunity strengthened (β = −0.212^**^) compared to the base model, as did the relations from opportunity to risk perception (β = −0.242^***^), from risk perception to possibility (0.250^**^), and from possibility to pro-environmental behavior (β = 0.494^***^). As a result, felt possibility to mitigate predicted pro-environmental behavior directly by 94%, and only 6% of its effect was mediated *via* felt responsibility (Appendix 4).

For *farmers under 40 years old*, **two** relations turned from negative to positive (from achievement to responsibility and to pro-environmental behavior) and one from positive to negative (from opportunity to responsibility). None of these relations were statistically significant. Statistical significance disappeared from value-based relations as in the model for women (from achievement to climate change belief, and from universalism to responsibility and to pro-environmental behavior). The direct effect from possibility to pro-environmental behavior (β = 0.234^***^) was lowest for younger farmers, but 10% its effect was mediated *via* responsibility (Appendix 4).

The model for *organic farmers* showed also differences to the basic model. The relations from achievement to climate change belief and from universalism to responsibility and to pro-environmental behavior lost their statistical significance. More strikingly, so did the relation from responsibility to pro-environmental behavior. However, the relations from universalism to climate change belief (β = 0.457^***^) and to possibility (β = 0.347^***^) strengthened compared to the base model. The direct effects of climate change belief to responsibility (β = 0.279^***^) and to pro-environmental behavior (β = 0.294^***^) were strongest for this farmer group, indicating the importance of universalism and climate change belief as more important predictors of pro-environmental action for organic farmers than for others.

## Discussion

Psychological research can give new and important insights into the research aiming at transition toward low-carbon economies (Swim et al., 2011; Clayton et al., 2015). The VBN theory (Stern, 2000) has been widely used to study the elements of pro-environmental behavior and has also been the basis for agricultural research (Price and Leviston, 2014; Sanderson et al., 2018). Climate change opportunities have been presented in the media and discussed in Finland (Peltonen-Sainio et al., 2018, 2020) and other boreal countries (King et al., 2018; Unc et al., 2021), but the effect of these opportunities to pro-environmental action has not been studied. In this research, we introduced a new element of climate change opportunities to the VBN theory structure to study its effects. We also experimented with separate models for different farmer groups and two different points in time.

Although this study contributes to the literature of VBN applications in farmer mitigation behavior, there is a set of limitations that should be considered when interpreting the results. First, the time-variant survey questionnaires were not designed homogeneously as regards to one statement (pro-environmental behavior), which prevented a full comparison of the predictive models. Thus, the comparison cannot be made at the model level, and only the other connections can be compared. Second, terminology used in different disciplines doing similar research is not coherent. This results in difficulties when trying to do transdisciplinary research and apply the methods to new sectors, such as agriculture. We have tried to address this issue in this research.

Our first hypothesis on the causal path from values to pro-environmental behavior according to the VBN theory was confirmed. The base model for 2020 predicted 30% of pro-environmental behavior within the farmer community and 29–40% was reached with the models for different farmer groups. Although the results from previous research were confirmed in many parts, there were also interesting differences. Starting from the *values*, unlike Sanderson et al. (2018), universalism was found to have both direct and indirect positive effects to all VBN theory elements, including pro-environmental behavior among the farming community (Figure 2; Table 2; Appendix 3), similar to the results of Price and Leviston (2014). Still, the effect was stronger, when it was mediated via the other theory elements, but direct effects were found from the 2020 base model and models for men, conventional farmers, and older farmers (Table 2). For women, organic, and younger farmers, the direct effect of universalism did not reach pro-environmental behavior, indicating that within these groups, a certain value base alone does not motivate direct pro-environmental behavior. The strongest direct connection from universalism was to felt possibility to influence agricultural emission in agriculture. This would indicate that pro-environmental values activate farmers to consider whether and how climate change could be addressed and find ways to mitigate these risks. The value of achievement, a self-enhancement value in the Schwartz et al. (2012) basic human values theory, was found to have a negative relation to climate change belief as reported in previous research (Schultz et al., 2005). Besides climate change belief, achievement is connected directly and positively to opportunity, but not to any other variables. In the model for women, the connections from achievement were not statistically significant to any of the other variables studied. This would imply that values having more personal focus do not impact pro-environmental behavior.

*Belief* of anthropogenic origins of climate change had a strong direct effect to risk perception and to felt possibility to mitigate (Figure 2). This connection has been repeatedly found in previous research, including in the agricultural sector (e.g., Niles, Lubell and Haden, 2013), and holds in it the idea that people can fix problems that they have created themselves (Böhm and Tanner, 2019). *Risk perception* acted as a mediator between the following VBN theory elements, but its direct effects were considerably lower than expected. Surprisingly, there was no direct effect from risk perception to pro-environmental behavior. Our results on risk perception confirm that the risks associated with climate change do not relate to everyday experiences that would lead to pro-environmental behavior (Leiserowitz, 2006; Weber, 2006; Haden et al., 2012) in the Finnish agricultural context.

The cause for the low importance of risk perception can be explained also through its connection to opportunities. Our second hypothesis on the negative effect of climate change *opportunities* to risk perception was confirmed indicating that the prospects of opportunities leave behind the possible risks that might occur in future. Here, biases and heuristics might also be relevant. Because of optimism bias, people tend to believe that positive events are more likely to happen to themselves and negative events to others (Weinstein, 1980). Affect heuristic, on the contrary, impacts through positive feeling (such as prospective opportunities) that result to risks being evaluated smaller than they actually are (Finucane et al., 2000). The role of biases and heuristics in pro-environmental behavior is interesting and would need more research.

Our hypothesis on negative effects of climate change opportunity had to be rejected in the case of felt *possibility* to mitigate climate change (Figure 2; Table 2). Here, the relation was strong and interestingly positive. What could be the connection between farmers believing in climate change opportunities and simultaneously believing in their possibilities to mitigate? This might originate from the same belief that humans are capable of solving man-made problems as was stated for the climate change beliefs' connection to possibilities. One idea could be that as the discussion in Finland has concentrated strongly on the possibilities of agriculture and farmers to mitigate climate change and the support offered to farmers for doing so (e.g., subsidies for carbon farming), this has strengthened both aspects, opportunities and possibilities. From this perspective, the opportunities of climate change come from the mitigation possibility that the farmers have and not from the direct effects of global warming. In the absence of previous research on climate change opportunities and its psychological effects to pro-environmental behavior, we look forward to the results of future studies to bring light into this interesting finding.

Felt possibility to mitigate climate change was the strongest predictor for pro-environmental behavior in all other groups studied, except young farmers (Table 2, Appendices 3, 4). Our results are similar with Price and Leviston (2014), who found that locus control (a form of self-efficacy) was the strongest predictor for pro-environmental behavior. This means that for farmers, it is most crucial to know that one can perform the pro-environmental behavior in practice.

This research has concentrated on farmers' mitigation behavior as a form of pro-environmental behavior. As the drivers for climate change adaptation in agriculture seem to differ from mitigation (Zhang et al., 2020; Sorvali et al., 2021), the connections between climate change opportunities and adaptation behavior should be studied further. As farmers are not a uniform group either based on their value profiles (Sorvali et al., 2022), their climate change views (Sorvali et al., 2021) nor the predictors of pro-environmental behavior studied here, more research should be focused on studying the differences between groups and the barriers and drivers for the different groups as regards to pro-environmental behavior. Young farmers would be one group of special interest as they will be the actors responsible for the future development of agriculture, and there seem to be fundamental differences in their thinking compared to the older farmer generation. The longitudinal aspect would also benefit from further research. The development of future environmental policy in the agricultural sector would benefit from the knowledge that which predictors of pro-environmental behavior change the most in time and what factors contribute to that change. Besides the research on the role of heuristics and biases effecting pro-environmental behavior mentioned earlier, there are still many other aspects to be considered. The role of emotions as an important predictor of pro-environmental behavior has been brought up in the literature concerning risk perception and decision-making under uncertainty (Brosch, 2021) and should also be studied in the context of agriculture and climate change mitigation. As farming is much more a lifestyle rooted in traditions and not just an occupation, emotions might help to predict also pro-environmental behavior better. Also, the literature on fair distribution of mitigation burden and farmers' views on climate justice has started to emerge (Puupponen et al., 2022) and that also might contribute to the VBN theoretical framework.

## Conclusion

Farmers are the only actors capable of mitigating climate change impacts in agriculture at the farm level, and the impact of their action is therefore crucial. As the possibility to mitigate proved to be of such crucial importance in predicting pro-environmental behavior, our results seem to confirm Stern (2000) hypothesis, where the more important behavior is in terms of its environmental impact, the less behavior is dependent on attitudinal variables, such as climate change belief or risk perception. Therefore, future efforts in agricultural research and policy should concentrate on bringing forth concrete farm-level mitigation practices with proven environmental benefits. This would be the most effective way to promote the low-carbon transition of agriculture.

## Data availability statement

The raw data supporting the conclusions of this article will be made available by the authors, without undue reservation.

## Ethics statement

Ethical review and approval was not required for the study on human participants in accordance with the local legislation and institutional requirements. The patients/participants provided their written informed consent to participate in this study.

## Author contributions

Conceptualization, writing—original draft preparation, and writing—review and editing: JS, XL, and JK. Methodology: JS. Formal analysis and investigation: JK. All authors contributed to the article and approved the submitted version.

## Funding

This work was funded by the European Commission Life-Program and Natural Resources Institute Finland (Luke) as a part of a consortium project called Optimizing Agricultural Land Use to Mitigate Climate Change (OPAL-Life, LIFE14 CCM/FI/000254; this paper only reflects the authors' view, and the EASME/Commission is not responsible for any use that may be made of the information it contains). This work was supported also by the Academy of Finland through DivCSA (Decision No. 316215) and by the Ministry of Agriculture and Forestry of Finland under the Catch the Carbon program (Project Tuima, VN/28648/2020-MMM-3).

## Conflict of interest

The authors declare that the research was conducted in the absence of any commercial or financial relationships that could be construed as a potential conflict of interest.

## Publisher's note

All claims expressed in this article are solely those of the authors and do not necessarily represent those of their affiliated organizations, or those of the publisher, the editors and the reviewers. Any product that may be evaluated in this article, or claim that may be made by its manufacturer, is not guaranteed or endorsed by the publisher.
