# Supplementary material for: Climate change opportunities reduce farmers' risk perception: Extension of the value-belief-norm theory in the context of Finnish agriculture
Source: Front Psychol. 2022 Aug 24;13:939201. doi: 10.3389/fpsyg.2022.939201 (PMC9449493; doi:10.3389/fpsyg.2022.939201)
Supplement: Supplementary file 1 [file Data_Sheet_1.PDF]

Appendix 1. Basic characteristics of the respondents and the total Finnish farming population. Data for the total farming population in Finland is from 2017 due to the lack of comparable data from 2018. All data from Natural Resources Institute Finland 2019 unless otherwise stated.

	**Finnish farmers 2017**		**Survey 2018**		**Survey 2020**	
	N	%	N	%	N	%
**Number of farms**	48,562		4,401		2,000	
**Gender**[1]						
**Female**	5,900	*12*	569	*13*	223	*11*
**Male**	43,820	*88*	3,831	*87*	1,777	*89*
**Age**						
**under 30**	1,376	*3*	137	*3*	12	*1*
**30-50**	15,214	*36*	1,844	*42*	719	*36*
**51-70**	23,343	*56*	2,289	*52*	1,167	*58*
**over 70**	1,945	*5*	129	*3*	98	*5*
**Education**[2]						
**Comprehensive**	8,741	*18*	325	*7*	138	*7*
**Vocational**	27,195	*56*	2,871	*65*	1,229	*62*
**University**	12,626	*26*	1,119	*25*	615	*31*
**Other**			84	*2*	18	*1*
**Farming system**						
**Organic**[3]	4,665	*10*	657	*15*	312	*16*
**Conventional**	43,897	*90*	3,743	*85*	1,688	*84*
**Farm size (ha)**						
**less than 30**	21,101	*44*	1,792	*41*	758	*38*
**30-49**	12,137	*25*	876	*20*	412	*21*
**50-99**	9,917	*20*	1,053	*24*	494	*25*
**100 and over**	5,407	*11*	679	*15*	302	*15*
**Revenue (euros)**[4]						
**less than 20 000**	23,592	*50*	886	*20*	418	*21*
**20 000-50 000**	9,359	*20*	1,111	*25*	520	*26*
**50 000-100 000**	5,939	*13*	914	*21*	382	*19*
**100 000-300 000**	6,385	*13*	1,032	*23*	469	*23*
**300 000 - 500 000**	1,101	*2*	280	*6*	116	*6*
**500 000 - 1 000 000**	676	*1*	176	*4*	67	*3*
**more than 1 000 000**	636	*1*	51	*1*	28	*1*
**Farm organization**						
**Family farm**	41,878	*86*	3,707	*84*	1,669	*85*
**Agricultural alliance**	4,178	*9*	433	*10*	195	*10*
**Limited liability company**	931	*2*	84	*2*	38	*2*
**Death estate**	1,227	*2*	93	*2*	35	*2*
**Other**	348	*1*	82	*2*	29	*1*
**Farm type**[5]						
**Cereals**	16,240	*33*	1893	*43*	844	*43*

**Special crops**	14,379	*30*	355	*8*	164	*8*
**Dairy production**	6,704	*14*	804	*18*	358	*18*
**Beef production**	3,485	*7*	287	*7*	129	*7*
**Horticultural production**	1,477	*3*	130	*3*	64	*3*
**Pig production**	607	*1*	165	*4*	87	*4*
**Poultry production**	436	*1*	65	*1*	30	*2*
**Greenhouse production**	836	*2*	11	*0 (0.3)*	8	*0 (0.4)*
**Other**	4,398	*9*	486	*11*	209	*11*
**Not known**	0	*0*	204	*5*	78	*4*
**Region[6]**						
Southern Finland	***14,809***	**31**	***1,471***	**35**	***684***	**34**
**Uusimaa**	3,173	*7*	313	*7*	*141*	*7*
**Southwest Finland**	5,175	*11*	537	*13*	*253*	*13*
**Southeast Finland**	2,957	*6*	277	*7*	*129*	*7*
**Häme**	3,504	*7*	344	*8*	*161*	*8*
Western Finland	***19,298***	**39**	***1,627***	**38**	***729***	**36**
**Satakunta**	2,976	*6*	334	*8*	*142*	*7*
**Pirkanmaa**	3,782	*8*	405	*10*	*183*	*10*
**Central Finland**	2,576	*5*	238	*6*	*100*	*5*
**South Ostrobothnia**	5,411	*11*	364	*9*	*171*	*9*
**Ostrobothnia**	4,553	*9*	286	*7*	*133*	*7*
Eastern Finland	***8,443***	**17**	***698***	**17**	***312***	**16**
**South Savo**	2,339	*5*	193	*5*	*92*	*5*
**North Savo**	3,448	*7*	295	*7*	*119*	*6*
**North Karelia**	2,009	*4*	151	*4*	*69*	*4*
**Kainuu**	647	*1*	59	*1*	*32*	*2*
Northern Finland	***5,609***	**12**	***399***	**9**	***164***	**8**
**North Ostrobothnia**	4,273	*9*	314	*7*	*131*	*7*
**Lapland**	1,336	*3*	85	*2*	*33*	*2*
Åland (i.e, archipelago)	***403***	**1**	***30***	**1**	***17***	**1**

[1](Eurostat, 2019). [2]Numbers for education are indicative due to limited data availability and differences in classification. [3](Finnish Food Authority, 2019). [4]Data for total of Finnish farmers from 2018. [5]Horticultural production included outdoor but not greenhouse production, whereas the group “others” consisted of farms sheep and horses, silviculture, honey production, landscape management activities, renting of land, tourism and contracting services. [6]Geographical division is based on the Eurostat NUTS 2003-2006 regions because it best corresponds to the administrative regional council structure in Finland and acknowledges traditional regional boundaries in Finland.
