# Supplementary material for: Climate change opportunities reduce farmers' risk perception: Extension of the value-belief-norm theory in the context of Finnish agriculture
Source: Front Psychol. 2022 Aug 24;13:939201. doi: 10.3389/fpsyg.2022.939201 (PMC9449493; doi:10.3389/fpsyg.2022.939201)
Supplement: Supplementary file 2 [file Data_Sheet_2.PDF]

Appendix 2. Questions measuring climate change belief, risk perception, opportunity, possibility, responsibility and pro-environmental behavior. Cronbach's alphas are given in parentheses, where applicable. Statements with marked with an asterisk (*) were measured with Likert-scale (1=Fully disagree, 2=Disagree, 3=Neither agree nor disagree, 4=Agree, 5=Fully agree).

Climate change belief *(Choose a statement that best describes your opinion)*
Climate change is not occurring
Not sufficient evidence to know whether climate change is occurring
Climate change is occurring, caused mainly by natural changes
Climate change is occurring, and it's caused equally by natural and human activities
Climate change is occurring, and it's mostly caused by human activities
Risk perception*
Climate change is a great threat to agriculture in Finland
Opportunity* *(α = .80)*
Climate change will be more beneficial than harmful to agriculture in Finland
Climate change creates new possibilities for Finnish agriculture
My farm will benefit from climate change
Finland's position as an important food producer will increase due to climate change
The yields will increase in Finland due to climate change
I will benefit economically from climate change
Possibility* *(α = .75)*
Farmer can mitigate climate change with farming practices
I can mitigate climate emissions at my own farm
My farming choices influence climate emissions
Practices made by the farmers can mitigate climate change in Finland
Climate actions taken by the agricultural sector significantly reduce Finland's climate emissions
Responsibility* *(α = .76)*
Agricultural sector must participate to climate change mitigation
I have to reduce climate emissions from my own farm
Pro-environmental behavior
I intend to mitigate climate change at my own farm (no, maybe, yes)
