# Supplementary material for: Climate change opportunities reduce farmers' risk perception: Extension of the value-belief-norm theory in the context of Finnish agriculture
Source: Front Psychol. 2022 Aug 24;13:939201. doi: 10.3389/fpsyg.2022.939201 (PMC9449493; doi:10.3389/fpsyg.2022.939201)
Supplement: Supplementary file 3 [file Data_Sheet_3.PDF]

Appendix 3. Total, direct and indirect effects (TE, DE and IE) of different variables and their statistical significance for all farmers in the 2020 base model. The percentage indicates the share of that effect in the total effect.

From	To	Effect	Estimate	SE	t value	p value	%
Achievement	CC Belief	TE	-0.092	0.022	-4.273	<.0001	
		DE	-0.092	0.022	-4.273	<.0001	100 %
		IE	0.000				0 %
Universalism	CC Belief	TE	0.269	0.021	12.895	<.0001	
		DE	0.269	0.021	12.895	<.0001	100 %
		IE	0.000				0 %
Achievement	Opportunity	TE	0.164	0.022	7.474	<.0001	
		DE	0.155	0.022	7.045	<.0001	94 %
		IE	0.009	0.003	3.069	0.002	6 %
Universalism	Opportunity	TE	-0.026	0.006	-4.166	<.0001	
		DE	0.000				0 %
		IE	-0.026	0.006	-4.166	<.0001	100 %
CC Belief	Opportunity	TE	-0.098	0.022	-4.421	<.0001	
		DE	-0.098	0.022	-4.421	<.0001	100 %
		IE	0.000				0 %
Achievement	Risk perception	TE	-0.081	0.010	-7.784	<.0001	
		DE	0.000				0 %
		IE	-0.081	0.010	-7.784	<.0001	100 %
Universalism	Risk perception	TE	0.162	0.021	7.912	<.0001	
		DE	0.089	0.021	4.337	<.0001	55 %
		IE	0.073	0.008	8.950	<.0001	45 %
CC Belief	Risk perception	TE	0.273	0.021	12.773	<.0001	
		DE	0.237	0.020	11.686	<.0001	87 %
		IE	0.036	0.008	4.333	<.0001	13 %
Opportunity	Risk perception	TE	-0.362	0.019	12.773	<.0001	
		DE	-0.362	0.019	12.773	<.0001	100 %
		IE	0.000				0 %
Achievement	Possibility	TE	-0.011	0.009	-1.272	0.203	
		DE	0.000				0 %
		IE	-0.011	0.009	-1.272	0.203	100 %
Universalism	Possibility	TE	0.325	0.020	16.246	<.0001	
		DE	0.214	0.020	10.762	<.0001	66 %
		IE	0.111	0.010	11.313	<.0001	34 %
CC Belief	Possibility	TE	0.350	0.020	17.876	<.0001	
		DE	0.318	0.020	15.834	<.0001	91 %
		IE	0.032	0.007	4.298	<.0001	9 %
Opportunity	Possibility	TE	0.135	0.020	6.837	<.0001	
		DE	0.203	0.021	9.772	<.0001	
		IE	-0.069	0.009	-7.811	<.0001	
Risk perception	Possibility	TE	0.189	0.022	8.681	<.0001	
		DE	0.189	0.022	8.681	<.0001	100 %
		IE	0.000				0 %

Achievement	Responsibility	TE	-0.049	0.020	-2.436	0.015	
		DE	-0.023	0.019	-1.181	0.238	46 %
		IE	-0.027	0.008	-3.297	0.001	54 %
Universalism	Responsibility	TE	0.286	0.021	13.829	<.0001	
		DE	0.105	0.020	5.222	<.0001	37 %
		IE	0.181	0.012	15.014	<.0001	63 %
CC Belief	Responsibility	TE	0.329	0.020	16.280	<.0001	
		DE	0.176	0.021	8.241	<.0001	53 %
		IE	0.153	0.011	13.387	<.0001	47 %
Opportunity	Responsibility	TE	0.024	0.021	1.177	0.239	
		DE	0.014	0.021	0.655	0.513	57 %
		IE	0.011	0.012	0.885	0.376	43 %
Risk perception	Responsibility	TE	0.173	0.023	7.654	<.0001	
		DE	0.105	0.022	4.826	<.0001	61 %
		IE	0.068	0.009	7.721	<.0001	39 %
Possibility	Responsibility	TE	0.360	0.021	17.168	<.0001	
		DE	0.360	0.021	17.168	<.0001	100 %
		IE	0.000				0 %
Achievement	Pro-environmental behavior	TE	-0.032	0.020	-1.569	0.117	
		DE	-0.002	0.019	-0.112	0.911	7 %
		IE	-0.030	0.008	-3.603	0.000	93 %
Universalism	Pro-environmental behavior	TE	0.276	0.021	13.244	<.0001	
		DE	0.088	0.020	4.301	<.0001	32 %
		IE	0.188	0.012	15.283	<.0001	68 %
CC Belief	Pro-environmental behavior	TE	0.319	0.021	15.585	<.0001	
		DE	0.160	0.022	7.310	<.0001	50 %
		IE	0.159	0.012	12.936	<.0001	50 %
Risk perception	Pro-environmental behavior	TE	0.053	0.023	2.264	0.024	
		DE	-0.036	0.022	-1.613	0.107	
		IE	0.088	0.010	8.675	<.0001	
Opportunity	Pro-environmental behavior	TE	0.017	0.021	0.818	0.414	
		DE	-0.046	0.021	-2.141	0.032	
		IE	0.063	0.012	5.178	<.0001	
Possibility	Pro-environmental behavior	TE	0.392	0.021	18.685	<.0001	
		DE	0.344	0.023	15.211	<.0001	88 %
		IE	0.048	0.009	5.589	<.0001	12 %
Responsibility	Pro-environmental behavior	TE	0.134	0.023	5.928	<.0001	
		DE	0.134	0.023	5.928	<.0001	100 %
		IE	0.000				0 %
