# Supplementary material for: Climate change opportunities reduce farmers' risk perception: Extension of the value-belief-norm theory in the context of Finnish agriculture
Source: Front Psychol. 2022 Aug 24;13:939201. doi: 10.3389/fpsyg.2022.939201 (PMC9449493; doi:10.3389/fpsyg.2022.939201)
Supplement: Supplementary file 4 [file Data_Sheet_4.PDF]

Appendix 4. Total, direct and indirect effects (TE, DE and IE) of different variables for different farmers groups. The percentage indicates the share of that effect in the total effect.

From	To	Effect	Women		Organic		Under 40	
			Estimate	%	Estimate	%	Estimate	%
Achievement	CC Belief	TE	-0.087		-0.051		-0.108	
		DE	-0.087	100 %	-0.051	100 %	-0.108	100 %
		IE	0	0 %	0	0 %	0	0 %
Universalism	CC Belief	TE	0.179		0.457		0.249	
		DE	0.179	100 %	0.457	100 %	0.249	100 %
		IE	0	0 %	0	0 %	0	0 %
Achievement	Opportunity	TE	0.059		0.193		0.161	
		DE	0.041	69 %	0.187	97 %	0.151	94 %
		IE	0.018	31 %	0.006	3 %	0.01	6 %
Universalism	Opportunity	TE	-0.038		-0.054		-0.023	
		DE	0	0 %	0	0 %	0	0 %
		IE	-0.038	100 %	-0.054	100 %	-0.023	100 %
CC Belief	Opportunity	TE	-0.212		-0.118		-0.091	
		DE	-0.212	100 %	-0.118	100 %	-0.091	100 %
		IE	0	0 %	0	0 %	0	0 %
Achievement	Risk perception	TE	-0.081		-0.081		-0.081	
		DE	0	0 %	0	0 %	0	0 %
		IE	-0.081	100 %	-0.081	100 %	-0.081	100 %
Universalism	Risk perception	TE	0.081		0.295		0.142	
		DE	0.04	50 %	0.176	60 %	0.078	55 %
		IE	0.041	50 %	0.119	40 %	0.065	45 %
CC Belief	Risk perception	TE	0.227		0.261		0.259	
		DE	0.137	60 %	0.224	86 %	0.224	87 %
		IE	0.09	40 %	0.037	14 %	0.034	13 %
Opportunity	Risk perception	TE	-0.424		-0.31		-0.375	
		DE	-0.424	100 %	-0.31	100 %	-0.375	100 %
		IE	0	0 %	0	0 %	0	0 %
Achievement	Possibility	TE	-0.027		0.004		-0.013	
		DE	0	0 %	0	0 %	0	0 %
		IE	-0.027	100 %	0.004	100 %	-0.013	100 %
Universalism	Possibility	TE	0.327		0.5		0.32	
		DE	0.257	79 %	0.347	69 %	0.223	70 %
		IE	0.07	21 %	0.153	31 %	0.097	30 %
CC Belief	Possibility	TE	0.335		0.258		0.328	
		DE	0.313	93 %	0.223	87 %	0.298	91 %
		IE	0.023	7 %	0.034	13 %	0.03	9 %
Opportunity	Possibility	TE	0.055		0.092		0.151	

		DE	0.161		0.154		0.224	
		IE	-0.106		-0.062		-0.074	
Risk perception	Possibility	TE	0.25		0.201		0.197	
		DE	0.25	100 %	0.201	100 %	0.197	100 %
		IE	0	0 %	0	0 %	0	0 %
Achievement	Responsibility	TE	-0.014		0.028		-0.072	
		DE	0.008	-59 %	0.056	198 %	-0.043	60 %
		IE	-0.023	159 %	-0.028	-98 %	-0.029	40 %
Universalism	Responsibility	TE	0.292		0.345		0.285	
		DE	0.17	58 %	0.033	10 %	0.117	41 %
		IE	0.122	42 %	0.312	90 %	0.167	59 %
CC Belief	Responsibility	TE	0.3		0.392		0.309	
		DE	0.214	72 %	0.279	71 %	0.169	55 %
		IE	0.085	28 %	0.113	29 %	0.14	45 %
Opportunity	Responsibility	TE	0.075		-0.042		0.028	
		DE	0.124	165 %	-0.022	53 %	0.021	74 %
		IE	-0.049	-65 %	-0.02	47 %	0.007	26 %
Risk perception	Responsibility	TE	0.205		0.203		0.184	
		DE	0.146	71 %	0.146	72 %	0.117	64 %
		IE	0.059	29 %	0.057	28 %	0.067	36 %
Possibility	Responsibility	TE	0.234		0.282		0.34	
		DE	0.234	100 %	0.282	100 %	0.34	100 %
		IE	0	0 %	0	0 %	0	0 %
Achievement	Pro-environmental behavior	TE	-0.061		-0.088		-0.052	
		DE	-0.04	66 %	-0.059	67 %	-0.017	31 %
		IE	-0.021	34 %	-0.029	33 %	-0.036	69 %
Universalism	Pro-environmental behavior	TE	0.274		0.362		0.279	
		DE	0.059	21 %	0.058	16 %	0.093	33 %
		IE	0.216	79 %	0.305	84 %	0.186	67 %
CC Belief	Pro-environmental behavior	TE	0.305		0.402		0.307	
		DE	0.127	42 %	0.294	73 %	0.153	50 %
		IE	0.178	58 %	0.108	27 %	0.154	50 %
Risk perception	Pro-environmental behavior	TE	0.053		-0.028		0.063	
		DE	-0.099		-0.112		-0.031	
		IE	0.152		0.085		0.094	
Opportunity	Pro-environmental behavior	TE	0.112		-0.075		0.014	
		DE	0.032	29 %	-0.136		-0.056	
		IE	0.08	71 %	0.061		0.07	
Possibility	Pro-environmental behavior	TE	0.526		0.355		0.406	
		DE	0.494	94 %	0.329	93 %	0.366	90 %

		IE	0.032	6 %	0.026	7 %	0.04	10 %
Responsibility	Pro-environmental behavior	TE	0.138		0.091		0.118	
		DE	0.138	100 %	0.091	100 %	0.118	100 %
		IE	0	0 %	0	0 %	0	0 %
